# Supplementary material for: Hybrid Models and Biological Model Reduction with PyDSTool
Source: PLoS Comput Biol. 2012 Aug 9;8(8):e1002628. doi: 10.1371/journal.pcbi.1002628 (PMC3415397; doi:10.1371/journal.pcbi.1002628)
Supplement: Text S4 — Complete source code for the PyDSTool package (version 0.88.120504). Includes API documentation and help files linking to web pages. This file is identical to the current public release on Sourceforge.net. (ZIP) [file pcbi.1002628.s004.zip › PyDSTool/html/PyDSTool.Interval'.Interval-class.html]

xml version="1.0" encoding="ascii"?


PyDSTool.Interval'.Interval


| Home | Trees | Indices | Help | PyDSTool |
| --- | --- | --- | --- | --- |

Package PyDSTool ::
Module Interval' ::
Class Interval

[hide private]

[frames] | no frames]

# Class Interval

source code

```
object --+
         |
        Interval
```

---

```
Numeric Interval class.

Numeric Interval implementation for integer and float types.

If the interval is not specified fully on initialisation then operations
    on the object are limited to set().
```


|  |  |
| --- | --- |
| Instance Methods | [hide private] |

|  |  |
| --- | --- |
| \_\_add\_\_(self, val) | source code |

|  |  |
| --- | --- |
| \_\_call\_\_(self) | source code |

|  |  |
| --- | --- |
| \_\_contains\_\_(self, val) | source code |

|  |  |
| --- | --- |
| \_\_copy\_\_(self) | source code |

|  |  |
| --- | --- |
| \_\_div\_\_(self, val) | source code |

|  |  |
| --- | --- |
| \_\_eq\_\_(self, other) | source code |

|  |  |
| --- | --- |
| \_\_ge\_\_(self, other) | source code |

|  |  |
| --- | --- |
| \_\_getitem\_\_(self, ix) | source code |

|  |  |
| --- | --- |
| \_\_getstate\_\_(self) | source code |

|  |  |
| --- | --- |
| \_\_gt\_\_(self, other) | source code |

|  |  |
| --- | --- |
| \_\_init\_\_(self, name, intervaltype, intervalspec=None, abseps=None)  x.\_\_init\_\_(...) initializes x; see x.\_\_class\_\_.\_\_doc\_\_ for signature | source code |

|  |  |
| --- | --- |
| \_\_le\_\_(self, other) | source code |

|  |  |
| --- | --- |
| \_\_lt\_\_(self, other) | source code |

|  |  |
| --- | --- |
| \_\_mul\_\_(self, val) | source code |

|  |  |
| --- | --- |
| \_\_ne\_\_(self, other) | source code |

|  |  |
| --- | --- |
| \_\_neg\_\_(self) | source code |

|  |  |
| --- | --- |
| \_\_radd\_\_(self, val) | source code |

|  |  |
| --- | --- |
| \_\_rdiv\_\_(self, val) | source code |

|  |  |
| --- | --- |
| \_\_repr\_\_(self)  str(x) | source code |

|  |  |
| --- | --- |
| \_\_rmul\_\_(self, val) | source code |

|  |  |
| --- | --- |
| \_\_rsub\_\_(self, val) | source code |

|  |  |
| --- | --- |
| \_\_setitem\_\_(self, ix, val) | source code |

|  |  |
| --- | --- |
| \_\_setstate\_\_(self, state) | source code |

|  |  |
| --- | --- |
| \_\_str\_\_(self)  str(x) | source code |

|  |  |
| --- | --- |
| \_\_sub\_\_(self, val) | source code |

|  |  |
| --- | --- |
| \_infostr(self, verbose=1)  Get info on a known interval definition. | source code |

|  |  |
| --- | --- |
| atEndPoint(self, val, bdcode)  val, bdcode -> Bool | source code |

|  |  |
| --- | --- |
| contains(self, val)  Report membership of val in the interval, returning type IntervalMembership. | source code |

|  |  |
| --- | --- |
| get(self, ix=None)  Get the interval as a tuple or a number (for singletons), or an endpoint if ix is not None | source code |

|  |  |
| --- | --- |
| info(self, verboselevel=1) | source code |

|  |  |
| --- | --- |
| intersect(self, other) | source code |

|  |  |
| --- | --- |
| isfinite(self) | source code |

|  |  |
| --- | --- |
| sample(self, dt, strict=False, avoidendpoints=False)  Sample the interval, returning a list. | source code |

|  |  |
| --- | --- |
| set(self, arg)  Define interval in an Interval object | source code |

|  |  |
| --- | --- |
| uniformSample(self, dt, strict=False, avoidendpoints=False)  Sample the interval, returning a list. | source code |

**Inherited from `object`**:
`__delattr__`,
`__getattribute__`,
`__hash__`,
`__new__`,
`__reduce__`,
`__reduce_ex__`,
`__setattr__`


|  |  |
| --- | --- |
| Properties | [hide private] |

**Inherited from `object`**:
`__class__`


|  |  |
| --- | --- |
| Method Details | [hide private] |

|  |  |
| --- | --- |
| \_\_init\_\_(self, name, intervaltype, intervalspec=None, abseps=None)  *(Constructor)* | source code |

x.\_\_init\_\_(...) initializes x; see x.\_\_class\_\_.\_\_doc\_\_ for
signature

Overrides: object.\_\_init\_\_: *(inherited documentation)*

|  |  |
| --- | --- |
| \_\_repr\_\_(self)  *(Representation operator)* | source code |

str(x)

Overrides: object.\_\_repr\_\_: *(inherited documentation)*

|  |  |
| --- | --- |
| \_\_str\_\_(self)  *(Informal representation operator)* | source code |

str(x)

Overrides: object.\_\_str\_\_: *(inherited documentation)*

|  |  |
| --- | --- |
| atEndPoint(self, val, bdcode) | source code |

val, bdcode -> Bool

Determines whether val is at the endpoint specified by bdcode, to the
precision of the interval's \_abseps tolerance. bdcode can be one of 'lo',
'low', 0, 'hi', 'high', 1

|  |  |
| --- | --- |
| sample(self, dt, strict=False, avoidendpoints=False) | source code |

```
Sample the interval, returning a list.

Arguments:

dt : sample step

strict : (Boolean) This option forces dt to be used throughout the interval,
  with a final step of < dt if not commensurate. Default of False
  is used for auto-selection of sample rate to fit interval
  (choice based on dt argument).

avoidendpoints : (Boolean, default False). When True, ensures that the first and
  last independent variable ("t") values are not included, offset by
  an amount given by self._abseps (the endpoint tolerance).
```

|  |  |
| --- | --- |
| uniformSample(self, dt, strict=False, avoidendpoints=False) | source code |

```
Sample the interval, returning a list.

Arguments:

dt : sample step

strict : (Boolean) This option forces dt to be used throughout the interval,
  with a final step of < dt if not commensurate. Default of False
  is used for auto-selection of sample rate to fit interval
  (choice based on dt argument).

avoidendpoints : (Boolean, default False). When True, ensures that the first and
  last independent variable ("t") values are not included, offset by
  an amount given by self._abseps (the endpoint tolerance).
```

  


| Home | Trees | Indices | Help | PyDSTool |
| --- | --- | --- | --- | --- |

|  |  |
| --- | --- |
| Generated by Epydoc 3.0.1 on Fri May 4 15:24:06 2012 | http://epydoc.sourceforge.net |
